# Supplementary material for: Dissecting the human leptomeninges at single-cell resolution
Source: Nat Commun. 2023 Nov 3;14:7036. doi: 10.1038/s41467-023-42825-y (PMC10624900; doi:10.1038/s41467-023-42825-y)
Supplement: Supplementary file 1 — Supplementary Information [file 41467_2023_42825_MOESM1_ESM.pdf]

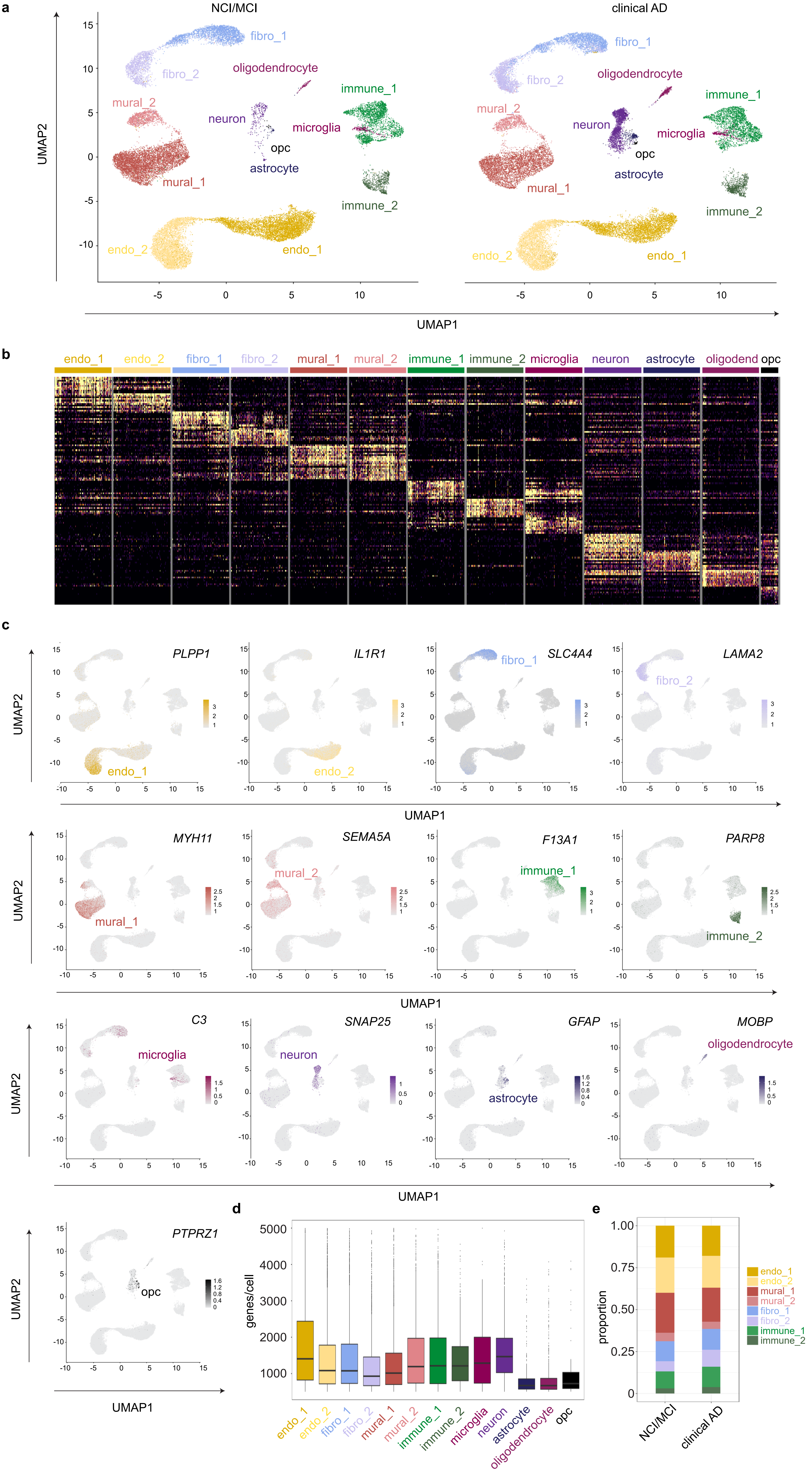

Supplementary Figure 1. snRNA-seq reveals major cell types of the human leptomeninges and adjacent parenchyma. (a) UMAP of meningeal and parenchymal cell types integrated across all donors, colored by coarse cell types, separated by NCI/MCI and AD. (b) Heatmap showing the top 10 markers per cell type across 100 randomly selected cells per cluster. (c) UMAP visualization of marker genes representing each cell cluster. (d) Boxplot shows the number of unique genes detected per nucleus in each cell type. (e) Stacked bar plot showing proportions of each cell subtype in leptomeningeal tissue of individuals with NCI/MCI and AD.

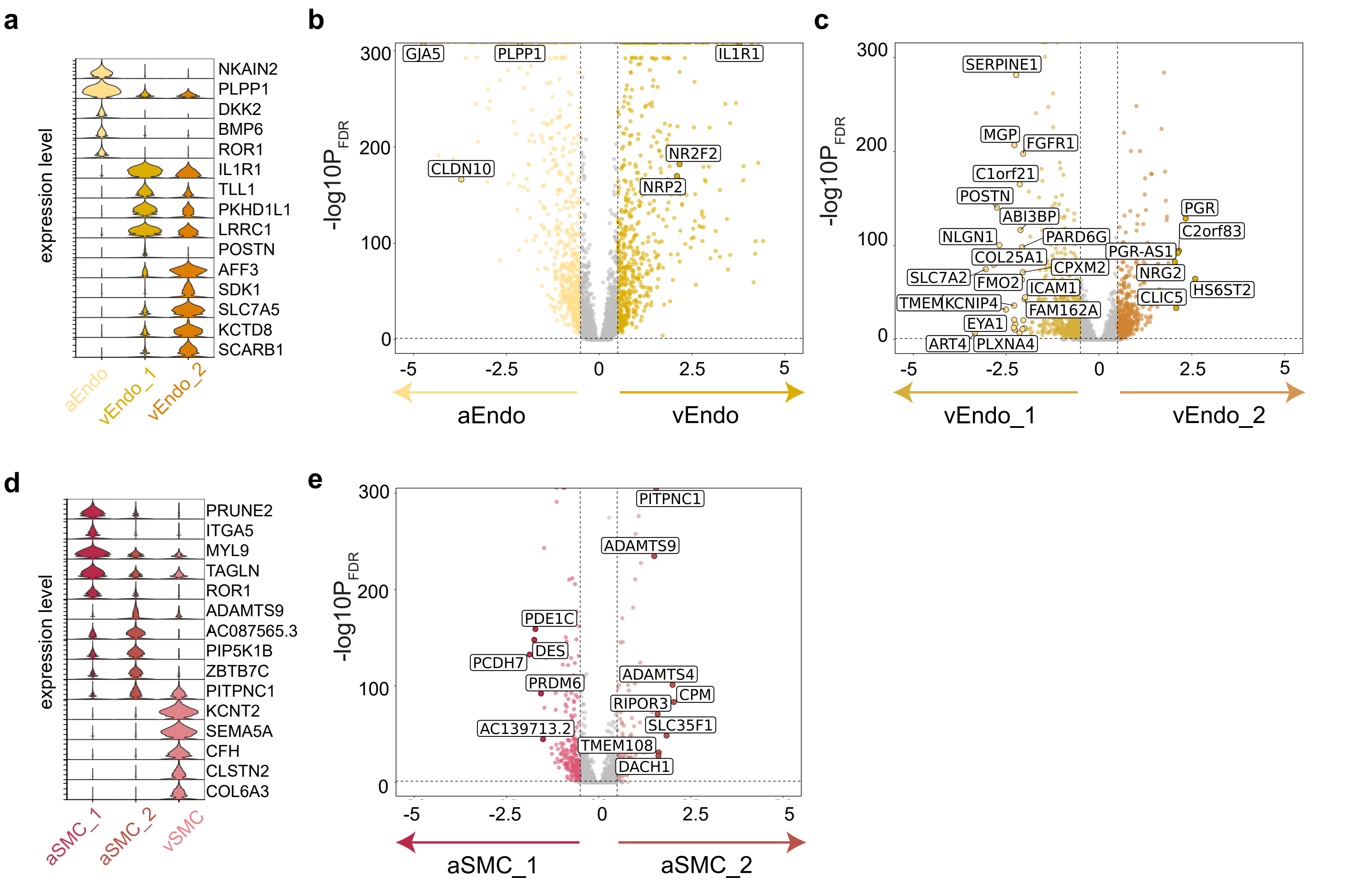

Supplementary Figure 2. Detailed transcriptomic profiles of the endothelial and smooth muscle cell subtypes. (a) Violin plot of the top 5 differentially expressed genes among endothelial subtypes. (b-c) Volcano plot of the differentially expressed genes distinguishing venous (vEndo) from arterial (aEndo) endothelial cells (b), and the two venous endothelial cell subtypes (c) with using a negative binomial generalized mixed model, Bonferroni correction; |logFC| > 0.5 and pBON < 0.01 (d) Violin plot of the top 5 differentially expressed genes among smooth muscle cell (SMC) subtypes. (e) Volcano plot of the differentially expressed genes distinguishing the two arterial SMC subtypes using a negative binomial generalized mixed model, Bonferroni correction; |logFC| > 0.5 and pBON < 0.01.

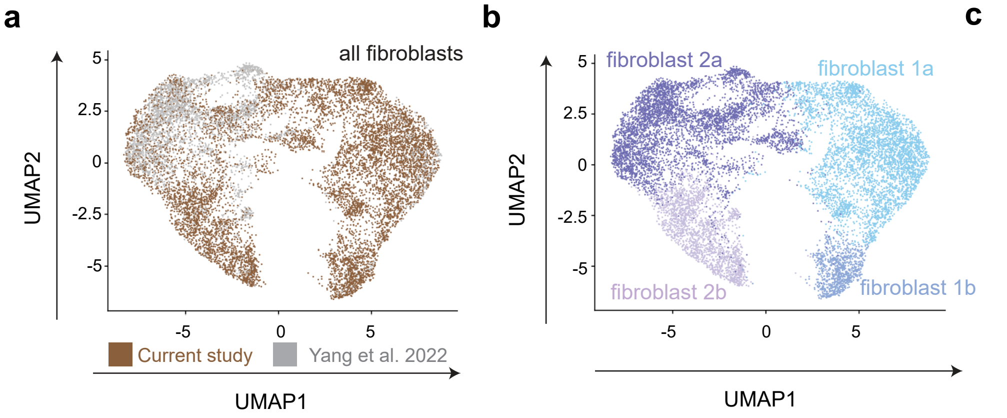

Supplementary Figure 3. Joint analysis of leptomeningeal and parenchymal fibroblast cells. (a-b) UMAP visualizations of integrated fibroblast subtypes from leptomeningeal (current study) and parenchymal (Yang et al., 2022), colored by study (a) or cell type (b). (c) Proportional representations of each subtype across each brain region by joint analysis.

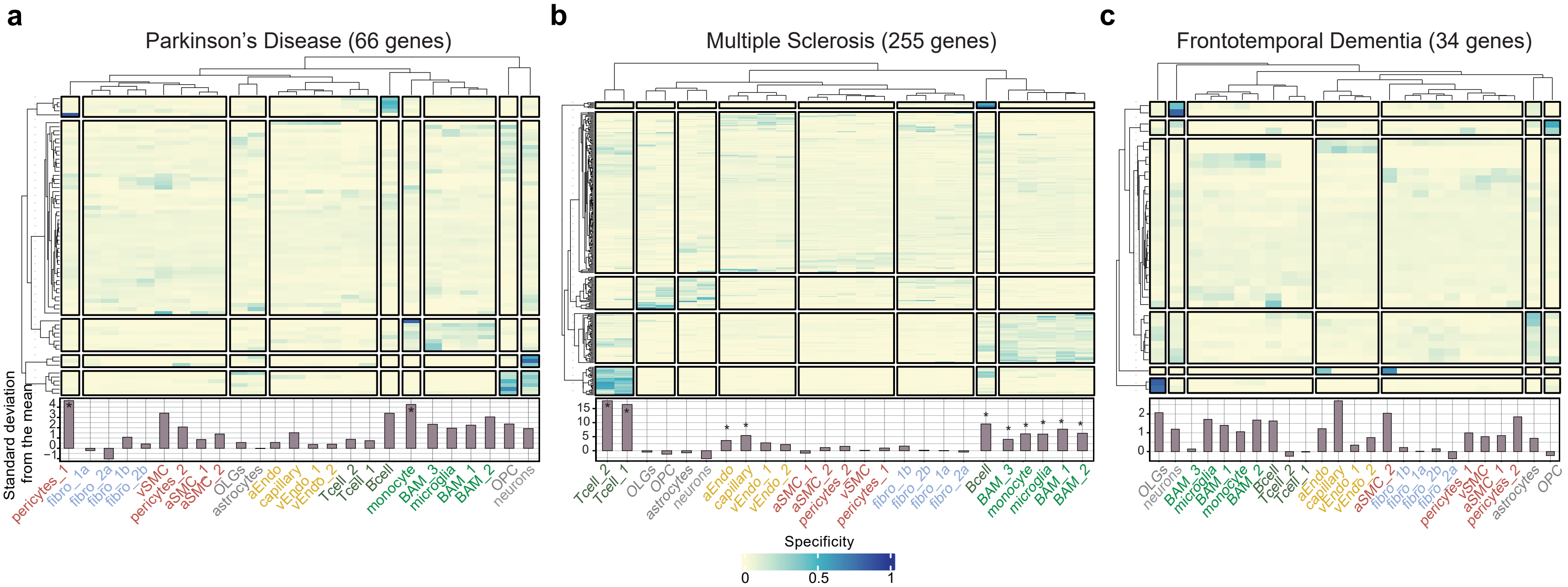

Supplementary Figure 4. Disease-specific GWAS gene expression in leptomeningeal cell types. (a-c) Heatmap of GWAS gene's proportional expression in each detected cell type across Parkinson's disease (a), Multiple sclerosis (b) and frontotemporal dementia (c). The values on the bottom represent the relative expression of all GWAS genes in each cell type. The barplots on the bottom show the standard deviation from the mean of a bootstrap test with 10,000 permutations as a proxy to the relative expression of all GWAS genes in each cell type. * denotes cell types with enriched expression for the respective GWAS genes.

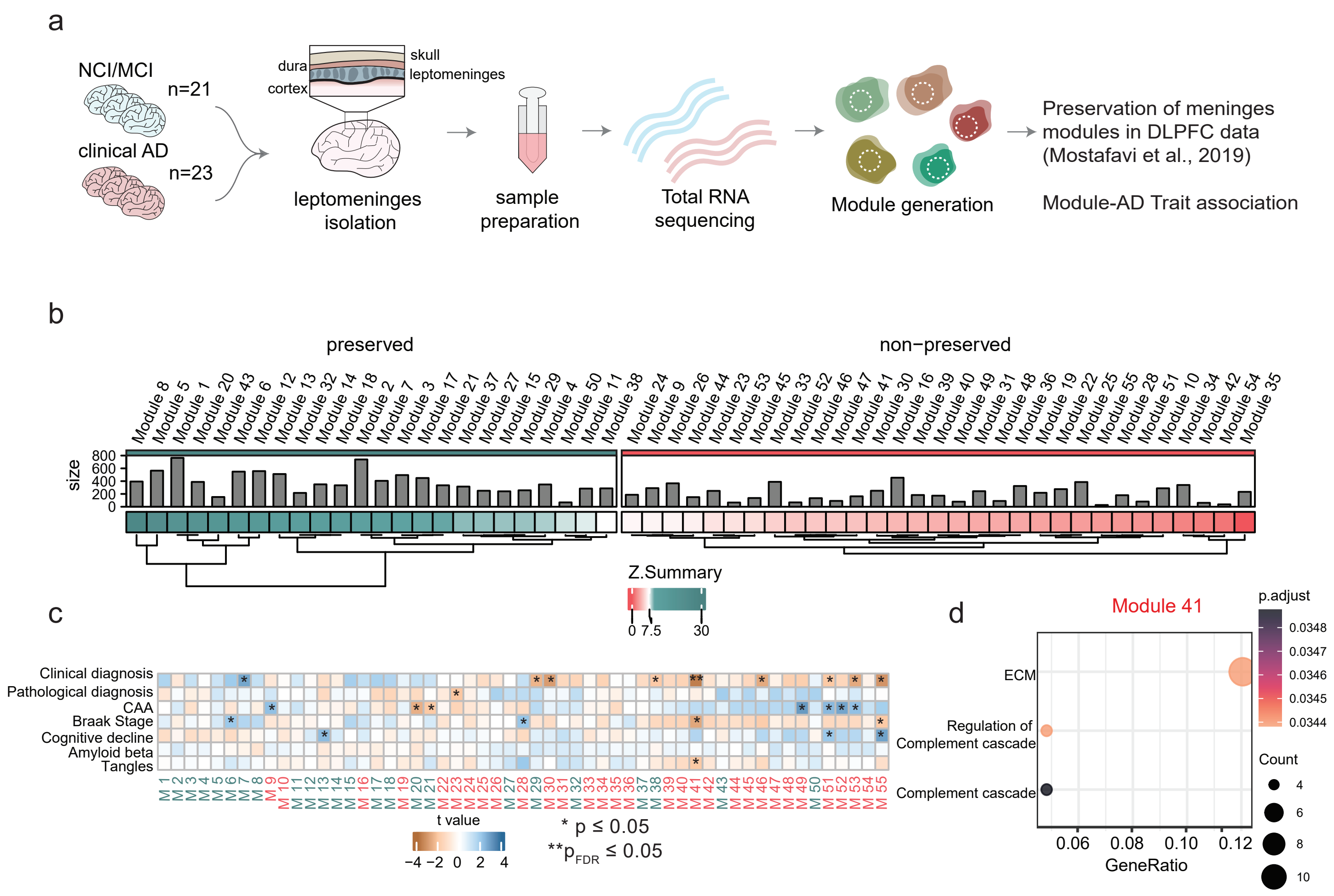

Supplementary Figure 5. Leptomeningeal gene co-expression modules and their association with AD traits. (a) Schematic illustration of experimental design, bulk RNA-seq and module-trait association analysis. (b) Co-expressed gene modules generated using SpeakEasy, and their gene set size (as bar plots) and preservation scores in the DLPFC data from Mostafavi et al., 2019. Modules with a preservation score (z.summary) lower than 7.5 are considered non-preserved. (c) Association between modules and AD-related traits, including clinical and pathological diagnosis, cerebral amyloid angiopathy (CAA), cognitive decline, plaques, and tangles (one-way ANOVA, Benjamini-Hochberg correction; *p ≤ 0.05, **pFDR ≤ 0.05). (d) Pathway analysis of the gene members of non-preserved module 41. Significant Reactome pathways are visualized (pBH < 0.05).

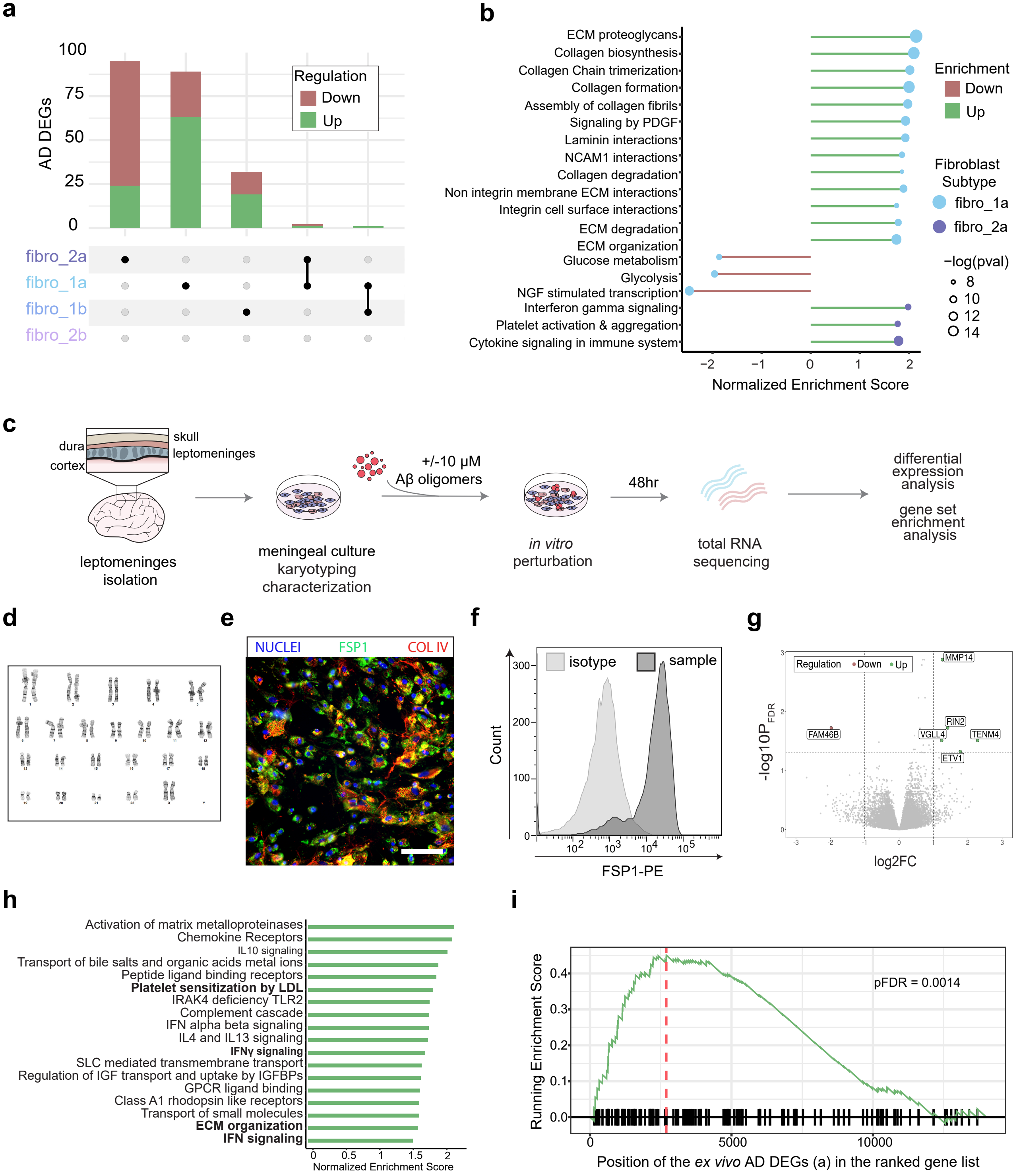

Supplementary Figure 6. Aβ-treated leptomeningeal cultures show the AD gene signature of ex vivo fibroblasts. (a) Differentially expressed genes (DEGs) between AD and control in each fibroblast subtype; negative binomial generalized mixed model, Bonferroni correction; threshold set at |logFC| > 0.3 and pBON < 0.01. (b) Gene set enrichment analysis reveals significant terms (PFDR < 0.05) for fibro_1a and 2a subtypes. (c) Schematic of leptomeningeal cell line derivation and Aβ treatment paradigm. (d) Representative karyotype result of a derived cell line. (e) Representative immunofluorescence images of cultured cells stained for nestin, collagen IV, and fibroblast-specific protein 1 (FSP1). Scale bar = 5μm. (f) Representative flow cytometry histogram showing the FSP1 expression of cultured fibroblasts. (g) DEGs in cultured fibroblasts upon Aβ treatment; linear regression model, Benjamini-Hochberg correction correction; |logFC| > 1 and pFDR < 0.5 are colored (h) Gene set enrichment analysis of the DEGs showing significant terms (PFDR < 0.05). (i) Enrichment score of the ex vivo fibroblast AD DEGs in the pre-ranked list of the in vitro Aβ-induced DEGs.

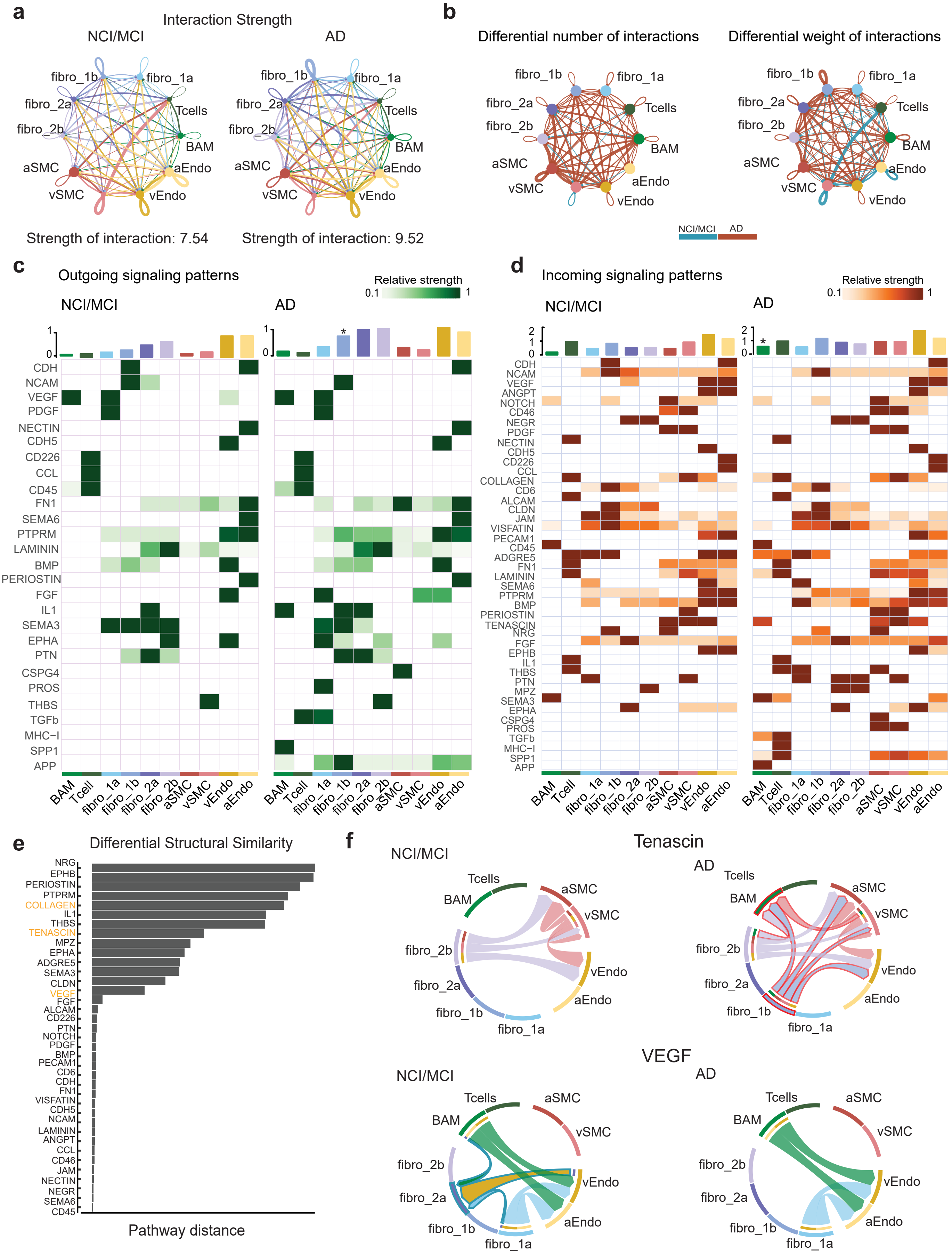

Supplementary Figure 7. Altered intercellular communications in AD leptomeninges. (a) Circular plots show the strength of inferred intercellular communications in control and AD groups. Autocrine (loops on top of each cell group) and paracrine (connections between cell groups) communications are displayed in each circular plot. (b) Differential number and weight of communications between the control and AD groups. Blue and red lines denote increased communications in control and AD, respectively. (c-d) Outgoing and incoming active signaling patterns across cell types in control and AD. The bar plots on the top show the sender or receiver's overall activities in each cell type, and the asterisks annotate significantly increased signaling in AD. Note that Fibro-1b and BAMs in AD display significantly increased outgoing and incoming activities, respectively, denoted by the black asterisk (one-sided Wilcoxon Rank-Sum $p < 0.05$) (e) Evaluation of similarity of signaling network topology of each shared pathway between the control and AD groups. (f) Circular plots show the altered Tenascin and VEGF pathways in AD. Differential interactions are highlighted in red for AD and blue for control.

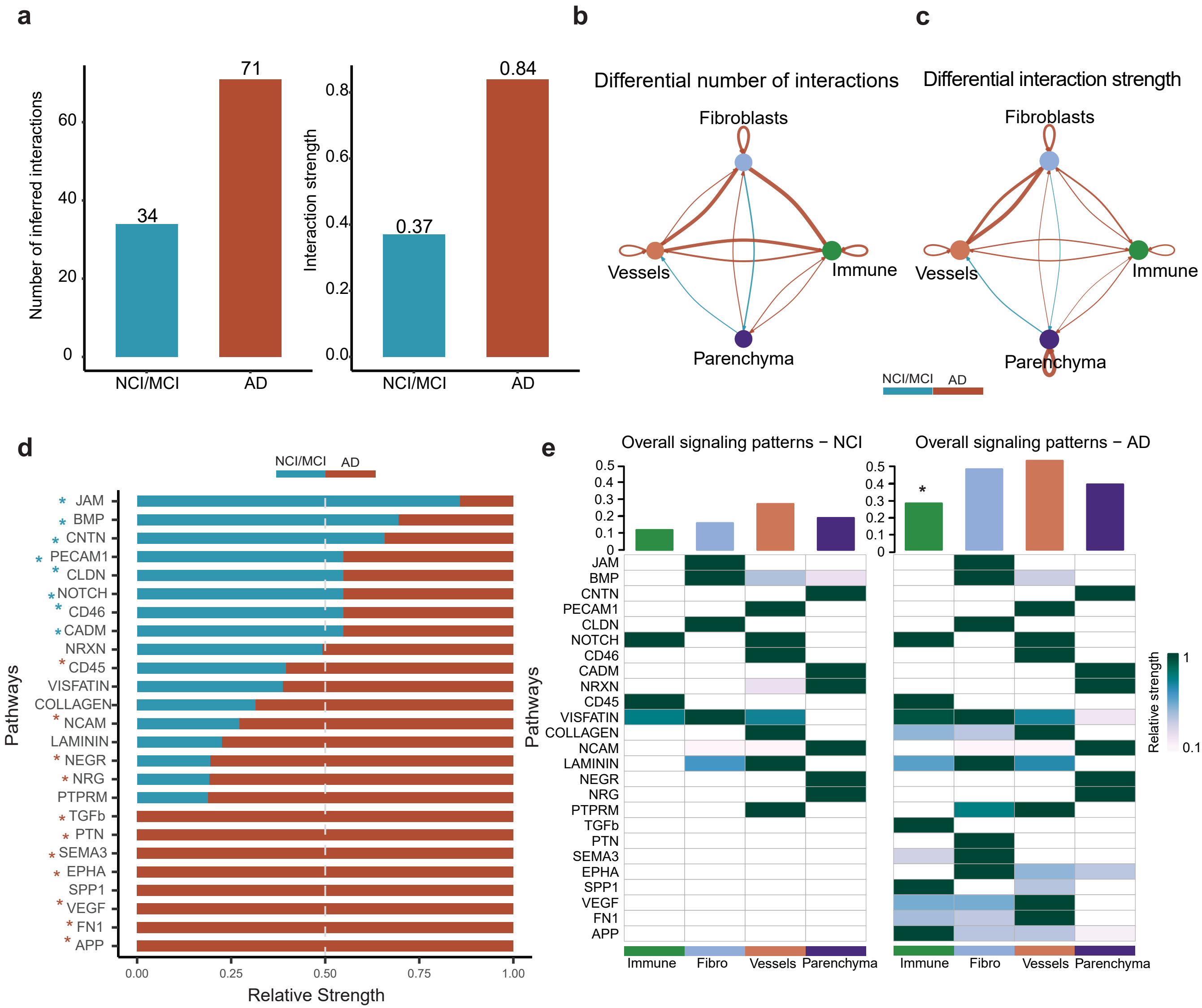

Supplementary Figure 8. Altered intercellular communications at AD leptomeninges-parenchyma interface. (a) Bar plots of the total number and strength of inferred interactions in control and AD. (b-c) Differential numbers and weight of interactions among parenchymal and leptomeningeal cells. Blue and red lines denote increased communications in control and AD, respectively. (d) The relative strength of 25 active signaling pathways at the leptomeninges-parenchyma interface in control and AD groups. Pathways annotated with blue and red stars are downregulated and upregulated in AD, respectively (two-sided Wilcoxon Rank-Sum $p < 0.05$) (e) The overall signaling patterns across cell types in NCI/MCI and AD. The bar plots on the top show the overall activity of each cell type. * denotes significantly increased activity.
