# Supplementary material for: Dissecting the human leptomeninges at single-cell resolution
Source: Nat Commun. 2023 Nov 3;14:7036. doi: 10.1038/s41467-023-42825-y (PMC10624900; doi:10.1038/s41467-023-42825-y)
Supplement: Supplementary file 3 — Description of Additional Supplementary Files [file 41467_2023_42825_MOESM3_ESM.pdf]

Description of Additional Supplementary Files

File Name: Supplementary Data 1
Description: Demographic characteristics of selected cases for ex vivo and in vitro experiments

File Name: Supplementary Data 2
Description: Proportional differences across cell types between NCI/MCI and AD. (two-tailed t-test, Benjamini-Hochberg correction). Related to Supplementary Figure 1.

File Name: Supplementary Data 3
Description: Marker genes for major cell types (two-sided Wilcoxon Rank Sum test, Bonferroni correction). Related to Figure 1 and Supplementary Figure 1.

File Name: Supplementary Data 4
Description: Differentially expressed genes for vascular cell types and trajectory analysis (negative binomial generalized mixed models, Bonferroni correction). Related to Figure 2.

File Name: Supplementary Data 5
Description: Marker genes for fibroblast subtypes (negative binomial generalized mixed models, Bonferroni correction). Related to Figure 3.

File Name: Supplementary Data 6
Description: Marker genes for T cell subtypes (negative binomial generalized mixed models, Bonferroni correction). Related to Figure 4.

File Name: Supplementary Data 7
Description: Differentially expressed genes between microglia and BAMs (negative binomial generalized mixed models, Bonferroni correction). Related to Figure 4.

File Name: Supplementary Data 8
Description: Marker genes for BAM clusters 1-3 (negative binomial generalized mixed models, Bonferroni correction). Related to Figure 5.

File Name: Supplementary Data 9
Description: Selected GWAS genes and their hierarchical clustering in Alzheimer's disease, Parkinson's disease, Multiple Sclerosis and Frontotemporal dementia. Related to Figure 5 and Supplementary Figure 4.

File Name: Supplementary Data 10
Description: Bulk RNA-seq Modules Related to Supplementary Figure 5.

File Name: Supplementary Data 11
Description: Module-trait association Related to Supplementary Figure 5.

File Name: Supplementary Data 12
Description: Differentially expressed genes in AD by cell types (negative binomial generalized mixed models, Bonferroni correction). Related to Supplementary Figure 6.

File Name: Supplementary Data 13
Description: Differentially expressed genes upon A-beta treatment (linear regression model, Benjamini-Hochberg correction). Related to Supplementary Figure 6.

File Name: Supplementary Data 14
Description: Primary antibodies and RNAscope probes. Related to Methods.
