# Supplementary material for: Dissecting the human leptomeninges at single-cell resolution
Source: Nat Commun. 2023 Nov 3;14:7036. doi: 10.1038/s41467-023-42825-y (PMC10624900; doi:10.1038/s41467-023-42825-y)
Supplement: Supplementary file 18 — Reporting Summary [file 41467_2023_42825_MOESM18_ESM.pdf]

Corresponding author(s): Yanling Wang
Last updated by author(s): Oct 1, 2023

Reporting Summary

Nature Portfolio wishes to improve the reproducibility of the work that we publish. This form provides structure for consistency and transparency in reporting. For further information on Nature Portfolio policies, see our Editorial Policies and the Editorial Policy Checklist.

Statistics

For all statistical analyses, confirm that the following items are present in the figure legend, table legend, main text, or Methods section.

n/a	Confirmed	
☐	☒	The exact sample size (*n*) for each experimental group/condition, given as a discrete number and unit of measurement
☒	☐	A statement on whether measurements were taken from distinct samples or whether the same sample was measured repeatedly
☐	☒	The statistical test(s) used AND whether they are one- or two-sided<br>*Only common tests should be described solely by name; describe more complex techniques in the Methods section.*
☐	☒	A description of all covariates tested
☐	☒	A description of any assumptions or corrections, such as tests of normality and adjustment for multiple comparisons
☐	☒	A full description of the statistical parameters including central tendency (e.g. means) or other basic estimates (e.g. regression coefficient) AND variation (e.g. standard deviation) or associated estimates of uncertainty (e.g. confidence intervals)
☐	☒	For null hypothesis testing, the test statistic (e.g. *F*, *t*, *r*) with confidence intervals, effect sizes, degrees of freedom and *P* value noted<br>*Give P values as exact values whenever suitable.*
☒	☐	For Bayesian analysis, information on the choice of priors and Markov chain Monte Carlo settings
☒	☐	For hierarchical and complex designs, identification of the appropriate level for tests and full reporting of outcomes
☒	☐	Estimates of effect sizes (e.g. Cohen's *d*, Pearson's *r*), indicating how they were calculated

Our web collection on statistics for biologists contains articles on many of the points above.

Software and code

Policy information about availability of computer code

Data collection	Sequencing data were collected with the Illumina NovaSeq6000 platform
Data analysis	STAR v2.4.2a, PicardTools v1.128, Kallisto v0.46, R v4.0.5, limma v3.46, clusterProfiler v4.1.4, WGCNA v1.70-3, SimplifyEnrichment v1.0, GOsemSim v1.30.2, GOsummaries v2.26, Cell Ranger v6.0.1, Seurat v4.2.0, Harmony v0.1.1, nebula v1.2.1, EnhancedVolcano v1.14.0, Monocle3 v, ClueGO v2.5.9, Cytoscape v3.9.1, ComplexUpset v1.3.3, fgsea v1.22.0, CellChat v1.4.0, QuPath v0.3.2, FlowJo v10,

For manuscripts utilizing custom algorithms or software that are central to the research but not yet described in published literature, software must be made available to editors and reviewers. We strongly encourage code deposition in a community repository (e.g. GitHub). See the Nature Portfolio guidelines for submitting code & software for further information.

Data

Policy information about availability of data

All manuscripts must include a data availability statement. This statement should provide the following information, where applicable:

- Accession codes, unique identifiers, or web links for publicly available datasets
- A description of any restrictions on data availability
- For clinical datasets or third party data, please ensure that the statement adheres to our policy

Sample information, gene modules, cluster maker genes, and differentially expressed genes are provided as Supplemental Data files. All bulk and snRNA-seq data are available at Synapse under accession code syn34512705 (https://www.synapse.org/#!Synapse:syn34512705). The data are available under controlled use

conditions set by human privacy regulations. To access the data, a data use agreement is required that ensures the anonymity of the ROSMAP study participants. A data use agreement can be agreed with either Rush University Medical Center (www.radc.rush.edu) or with SAGE, which maintains Synapse.

Human research participants

Policy information about studies involving human research participants and Sex and Gender in Research.

Reporting on sex and gender	The scope of this study is to characterize the cell types in the leptomeninges and to test differences between demented and non-demented controls, so while we included individuals from both sexes in case selection, we did not conduct sex-based analyses. Aggregate sex can be found in Supplementary Data 1, sex for each sample is available with the raw data on Synapse.
Population characteristics	44 leptomeningeal samples were used for RNA-seq and grouped based on clinical diagnosis of Alzheimer's dementia (n=23) and no cognitive impairment (NCI) or mild cognitive impairment (MCI) (n=21) with age (92.5 +/- 7.69 and 91.35 +/- 5.12), and sex (34.8% and 23.8% male) as covariates. A subset of 18 samples were used for snRNA-seq and grouped based on clinical diagnosis of Alzheimer's dementia (n=9) and no cognitive impairment (NCI) or mild cognitive impairment (MCI) (n=9) with age (93.1 +/- 4.36 and 93.4 +/- 4.27), and sex (44.4% and 44.4% male) as covariates. Aggregated data for additional characteristics not used as covariates can be found in Supplementary Data 1.
Recruitment	This study used brain tissue previously collected as part of the ROSMAP study - participants were not specifically recruited for this study.
Ethics oversight	Human leptomeninges tissue was collected from 46 individuals from the Religious Orders Study or Rush Memory and Aging Project (ROSMAP) study. Both studies were approved by an Institutional Review Board (IRB) of Rush University Medical Center and in accordance with the criteria set by the Declaration of Helsinki. All participants signed an informed consent and Anatomic Gift Act.

Note that full information on the approval of the study protocol must also be provided in the manuscript.

Field-specific reporting

Please select the one below that is the best fit for your research. If you are not sure, read the appropriate sections before making your selection.

☒ Life sciences ☐ Behavioural & social sciences ☐ Ecological, evolutionary & environmental sciences

For a reference copy of the document with all sections, see nature.com/documents/nr-reporting-summary-flat.pdf

Life sciences study design

All studies must disclose on these points even when the disclosure is negative.

Sample size	For ex vivo tissue population sequencing we aimed to analyse at least 20 specimens per group with a comparable distribution of females (~75%) and matched for age, and post-mortem interval. Our final dataset therefore included 21 individuals with mild cognitive impairment or no clinical diagnosis of AD, and 23 individuals with clinical diagnosis of AD.<br><br>For single nuclei sequencing, we aimed to collect an average of 1000-2000 nuclei per individual from 18 individuals to obtain a dataset of over 36,0000 nuclei, as comparable studies have shown that similar or smaller sample sizes are sufficient to identify transcriptionally distinct cell types in post mortem human brain regions (https://doi.org/10.1038/s41586-022-04521-7 (17K) and https://doi.org/10.1038/s41586-022-04521-7 {10-20K per region)).
Data exclusions	For bulk RNA sequencing, we restricted tissue to those with PMI < 10 hours. Any samples failing genotype matching to the corresponding brain tissue were also excluded from selection. Post processing, samples were excluded if the total reads mapped were less than 5 million. For single nuclei data we excluded any droplets from the Cell Ranger outputs containing <500 or >5000 genes and/or> 5% mitochondrial RNA reads. Cells with transcriptional signatures of more than one cluster were excluded. For cell type characterization, following data integration, cell type and subtype clusters were inspected for contribution proportions by participant. We excluded the cells from one immune cluster that expressed markers of neutrophils as it was exclusively present in a single participant and therefore could not be replicated across participants. For in vitro experiments, we derived cell lines from many participants but excluded any that displayed clonal abnormalities following karyotyping.
Replication	Verification of the single-nuclei data findings was performed using RNAScope and immunohistochemistry on post mortem brain tissue. These experiments supported the cell type cluster findings. Data are presented in figures. All attempts at replication were successful.
Randomization	For both bulk and single nuclei analyses participants were grouped into high and low cognition groups using clinical diagnosis. The groups were balanced for age, sex and cerebral amyloid angiopathy load. For cell line experiments, all lines without clonal abnormalities were used.
Blinding	Investigators were blinded to group allocations during autopsy, tissue processing, sequencing library generation and initial data preprocessing and analysis in addition to cell line generation.

Reporting for specific materials, systems and methods

We require information from authors about some types of materials, experimental systems and methods used in many studies. Here, indicate whether each material, system or method listed is relevant to your study. If you are not sure if a list item applies to your research, read the appropriate section before selecting a response.

Materials & experimental systems

n/a	Involved in the study
☐	☒ Antibodies
☐	☒ Eukaryotic cell lines
☒	☐ Palaeontology and archaeology
☒	☐ Animals and other organisms
☒	☐ Clinical data
☒	☐ Dual use research of concern

Methods

n/a	Involved in the study
☒	☐ ChIP-seq
☐	☒ Flow cytometry
☒	☐ MRI-based neuroimaging

Antibodies

Antibodies used

ACTA2 antibody ThermoFisher 14-9760-82, dilution 1:1000
PECAM antibody Novus NB100-2284, dilution 1:50
Factor XIIIa antibody Novus NBP1-83938, dilution 1:200
HLA-DR antibody Fisher MSM2-3475-P1, dilution 1:100
CD8 antibody Fisher MA5-14548, dilution 1:100
CD45 antibody Leica LCA-L-CE, dilution 1:50
DCN antibody ThermoFisher PA5-13538, dilution 1:100
NG2 (CSPG4) antibody Novus NBP1-94154, dilution 1:1000
Collagen IV antibody Millipore Sigma AB769, dilution 1:100
FSP1 antibody Millipore Sigma SAB4200821, dilution 1:100
PE anti-human S100A4 antibody Biolegend 370004, dilution 1:100

Validation

ACTA2 antibody, validated by the company for Human in Western Blot, IHC and ICC/IF
PECAM antibody, validated by the company for Human in Western Blot, IHC and ICC/IF
Factor XIIIa antibody, validated by the company for Human in IHC
HLA-DR antibody, validated by the company for Human in Western Blot, IHC, ICC/IF and flow
CD8 antibody, validated by the company for Human in IHC and flow
CD45 antibody, validated by the company for Human in IHC
DCN antibody, validated by the company for Human in Western Blot, IHC, and flow
NG2 (CSPG4) antibody, validated by the company for Human in IHC
Collagen IV antibody, validated by the company for Human in ICC/IF and ELISA
FSP1 antibody, validated by the company for Human in Western Blot, IHC, ICC/IF and flow
PE anti-human S100A4 antibody, validated by the company for Human in flow

Eukaryotic cell lines

Policy information about cell lines and Sex and Gender in Research

Cell line source(s)	Cell lines were derived from leptomeningeal tissue from 4 male and 8 female donors. Phenotype information is aggregated in Supplementary Data 1 and available with raw data on Synapse.
Authentication	Cell lines were genotyped and karyotyped to confirm identity
Mycoplasma contamination	The cell lines were tested for mycoplasma testing using Lonza MycoAlert Mycoplasma detection kit performed according to the manufacturer's protocol. All results were negative for mycoplasma.
Commonly misidentified lines (See ICLAC register)	n/a

Flow Cytometry

Plots

Confirm that:

☒ The axis labels state the marker and fluorochrome used (e.g. CD4-FITC).

☒ The axis scales are clearly visible. Include numbers along axes only for bottom left plot of group (a 'group' is an analysis of identical markers).

☐ All plots are contour plots with outliers or pseudocolor plots.

☐ A numerical value for number of cells or percentage (with statistics) is provided.

Methodology

Sample preparation	Cell lines derived from post mortem leptomeninges tissue were washed with PBS and singularized using trypLE. Cells were resuspended in a flow buffer (PBS+ 0.5% BSA) and counted. Cells were blocked and incubated with FSPl antibody on ice for 30 mins. Cells were washed with buffer and resuspended in buffer containing 7AAD, samples were incubated a further 15 minutes before acquisition on a flow cytometer.
Instrument	Sony SH800
Software	FlowJo v10
Cell population abundance	n/a
Gating strategy	Cells were gated as 7AAD- on a 7AAD/FSC plot and doublets excluded with a FSC-A/FSC-H plot. An isotype control was used to gate positive cells

☐ Tick this box to confirm that a figure exemplifying the gating strategy is provided in the Supplementary Information.
